# 4145 Stepping Stones for Success in T3-T4 Translation: Building Collective Impact

**DOI:** 10.1017/cts.2020.428

**Published:** 2020-07-29

**Authors:** Kishore Athreya, Terry Nakazono, Jim Morrison, Pamela Davidson

**Affiliations:** 1 David Geffen School of Medicine at UCLA; 2 UCLA Clinical and Translational Science Institute

## Abstract

OBJECTIVES/GOALS: We will investigate the influence of multisector partnerships in T3-T4 research associated with advances in delivery systems, patient/population outcomes and health policy and the translational processes linked to these improvements. METHODS/STUDY POPULATION: We are using both quantitative and qualitative data to measure and analyze partnership characteristics linked to successful translation into practice & policy. We aim to complete 100 surveys of investigators who have conducted CTSA-supported T3-T4 research to examine partnerships, conditions of collective impact, and quantifiable changes in delivery systems, health outcomes, and policy. Using rigorous criteria, we will select projects for more in-depth interviews to understand the practices of successful translation and roadblocks and barriers that challenge translation. RESULTS/ANTICIPATED RESULTS: The anticipated research products include: (i) an analytic report on partnership structure and processes and the statistical associations to stages of change outcomes, (ii) a series of vignettes to describe the impact stories and translational processes, (iii) cross-project analysis of the data and vignettes to produce generalizable information to improve T3-T4 translation, and (iv) peer-reviewed manuscript(s) for publication. DISCUSSION/SIGNIFICANCE OF IMPACT: The study will inform and improve researcher competencies and accelerate translation in CTSA hubs that emphasize T3-T4 research. We will develop novel definitions of the T3-T4 research impact. Ultimately, the results will inform research training to better address real-world priorities and needs.

